# Antenatal treatment with corticosteroids for preterm neonates: impact on the incidence of respiratory distress syndrome and intra-hospital mortality

**DOI:** 10.1590/S1516-31802003000200003

**Published:** 2003-03-05

**Authors:** Joice Fabíola Meneguel, Ruth Guinsburg, Milton Harumi Miyoshi, Clovis de Araujo Peres, Regina Helena Russo, Benjamin Israel Kopelman, Luiz Camano

**Keywords:** Newborn, Infant, Respiratory, Distress, Syndrome, Antenatal, Corticosteroids, Recém, Nascido, Prematuro, Síndrome, Desconforto, Respiratório, Corticosteróide, Antenatal

## Abstract

**CONTEXT:**

Although the benefits of antenatal corticosteroids have been widely demonstrated in other countries, there are few studies among Brazilian newborn infants.

**OBJECTIVE:**

To evaluate the effectiveness of antenatal corticosteroids on the incidence of respiratory distress syndrome and intra-hospital mortality among neonates with a gestational age of less than 34 weeks.

**TYPE OF STUDY:**

Cross-sectional.

**SETTING:**

A tertiary-care hospital.

**PARTICIPANTS:**

Neonates exposed to any dose of antenatal corticosteroids for fetal maturation up to 7 days before delivery, and newborns paired by sex, birth weight, gestational age and time of birth that were not exposed to antenatal corticosteroids. The sample obtained consisted of 205 exposed newborns, 205 non-exposed and 39 newborns exposed to antenatal corticosteroids for whom it was not possible to find an unexposed pair.

**PROCEDURES:**

Analysis of maternal and newborn records.

**MAIN MEASUREMENTS:**

The primary clinical outcomes for the two groups were compared: the incidence of respiratory distress syndrome and intra-hospital mortality; as well as secondary outcomes related to neonatal morbidity.

**RESULTS:**

Antenatal corticosteroids reduced the occur-rence of respiratory distress syndrome (OR: 0.33; 95% CI: 0.21-0.51) and the protective effect persisted when adjusted for weight, gestational age and the presence of asphyxia (adjusted OR: 0.27; 95% CI: 0.17-0.43). The protective effect could also be detected through the reduction in the need for and number of doses of exogenous surfactant utilized and the number of days of mechanical ventilation needed for the newborns exposed to antenatal corticosteroids. Their use also reduced the occurrence of intra-hospital deaths (OR: 0.51: 95% CI: 0.38-0.82). However, when adjusted for weight, gestational age, presence of prenatal asphyxia, respiratory distress syndrome, necrotizing enterocolitis and use of mechanical ventilation, the antenatal corticosteroids did not maintain the protective effect in relation to death. With regard to other outcomes, antenatal corticosteroids reduced the incidence of intraventricular hemorrhage grades III and IV (OR: 0.28; 95% CI: 0.10-0.77).

**CONCLUSIONS:**

Antenatal corticosteroids were effective in the reduction of morbidity and mortality among premature newborns in the population studied, and therefore their use should be stimulated within our environment.

## RESUMO

**CONTEXTO:**

Apesar de os benefícios da corticoterapia antenatal estarem amplamente demonstrados nos países desenvolvidos, há poucos estudos envolvendo o uso do corticosteróide antenatal nos neonatos brasileiros.

**OBJETIVOS:**

Avaliar a efetividade do corticosteróide antenatal na evolução de recémnascidos prematuros com idade gestacional inferior a 34 semanas.

**TIPO DE ESTUDO:**

Estudo retrospectivo.

**LOCAL:**

Hospital de atendimento terciário.

**PARTICIPANTES:**

Recém-nascidos expostos a qualquer dose de corticosteróide antenatal para maturação fetal até sete dias antes do parto e recém-nascidos pareados pelo sexo, peso ao nascer, idade gestacional e época de nascimento não expostos ao corticosteróide. Obteve-se uma amostra de 205 expostos, 205 controles e 39 recém-nascidos expostos ao corticosteróide antenatal para os quais não foi possível encontrar um par não exposto.

**PROCEDIMENTOS:**

Análise dos prontuários maternos e dos recém-nascidos.

**VARIÁVEIS ESTUDADAS:**

Compararam-se os desfechos clínicos primários: incidência de síndrome do desconforto respiratório e de óbito intra-hospitalar nos dois grupos, além de desfechos secundários relacionados à morbidade neonatal.

**RESULTADOS:**

O corticosteróide antenatal reduziu a ocorrência da síndrome do desconforto respiratório (razão de chances, OR:0,33; intervalo de confiança, IC, 95%:0,21-0,51) e o efeito protetor persistiu quando ajustado para o peso, idade gestacional e presença de asfixia (OR ajustada:0,27; IC 95%: 0,17-0,43). O efeito protetor pôde ser detectado também através da redução da necessidade e do número de doses de surfactante exógeno utilizadas e do número de dias de ventilação mecânica nos recém-nascidos expostos à corticoterapia antenatal. O corticosteróide antenatal, quando analisado isoladamente, reduziu a ocorrência do óbito intra-hospitalar (OR 0,51; IC 95%:0,38-0,82). No entanto, quando ajustados para o peso, a idade gestacional, a presença de pré-natal, asfixia, síndrome do desconforto respiratório, enterocolite necrosante e uso de ventilação mecânica, o corticosteróide antenatal não exerceu efeito protetor em relação ao óbito. Quanto aos outros desfechos, o corticosteróide desempenhou papel protetor para a hemorragia peri-intraventricular grave (OR:0,28; IC 95%:0,10-0,77).

**CONCLUSÕES:**

O corticosteróide antenatal foi efetivo para a redução da morbimortalidade de recém-nascidos prematuros na população do estudo e o seu uso deve ser estimulado no nosso meio.

## INTRODUCTION

Prematurity represents a serious problem for healthcare services throughout the world. Respiratory distress syndrome continues to be the most important pulmonary problem during the neonatal period, affecting a large number of premature infants. In Brazil, respiratory distress syndrome together with other respiratory problems was responsible for 44% of deaths due to perinatal affections in the year 1990, and this number increased to 49% in 1995. Despite the widespread use of exogenous surfactants for respiratory distress syndrome, mortality caused by this respiratory affection in Brazilian intensive care units continues to be four to five times greater than in developed countries.^1^

There are some relatively simple obstetric procedures for preventing the neonatal complications that lead to increases in morbidity, mortality and sequelae among survivors. The use of antenatal corticosteroids for fetal maturation is one of these procedures.^2^

The initial studies on antenatal corticosteroids had their scientific grounding in investigations on animals done in the 1960s. In these, it was noted that glucocorticoids were capable of accelerating the development process of organs and systems, especially for the lungs.^3^ In 1972, Liggins & Howie^4^ demonstrated a reduction in the incidence of respiratory distress syndrome among human neonates that received antenatal corticosteroids, and a reduction in their mortality. After this classic study, many others were made.^5-16^ Nevertheless, these studies were not enough to stimulate the routine use of antenatal corticosteroids in obstetric practice. This situation persisted until 1990, when Crowley et al.^17^ brought together 12 controlled studies of good methodological quality in a meta-analysis. These studies involved the use of antenatal corticosteroids or placebo on around 3,000 patients, and the meta-analysis demonstrated that antenatal medication promoted a 50% reduction in the incidence of respiratory distress syndrome and 40% in neonatal mortality, as well as a reduction in the occurrence of peri-intraventricular hemorrhage in the newborns treated. Soon afterwards, in 1994, the National Institutes of Health brought together several specialists involved in perinatology and established a consensus^18^ on the use of antenatal corticosteroids. This was an attempt to stimulate and disseminate the use of antenatal steroids in clinical practice.

Corticosteroids interact with specific receptor proteins in the target tissue so as to regulate the expression of the genes that are responsive to corticosteroids. Thus, the levels and disposition of the proteins synthesized by the various target tissues are altered.^19^ In this way, corticosteroids assist in achieving a successful transition from fetal to extra-uterine life, accelerating fetal maturation as a whole.^20-27^ The acceleration of lung development leads to a reduction in the incidence of respiratory distress syndrome and its severity.^17,28-30^ The structural and biochemical alterations induced by corticosteroids translate into a diminution of the need for and duration of mechanical ventilation for newborns with respiratory insufficiency.^31-33^ In developed countries, a significant reduction has also been observed in deaths from respiratory diseases through the widespread use of antenatal corticosteroids.^34^ This antenatal therapy should be considered not only because of the pulmonary maturation, but also for its protective role in the premature infant's brain. The use of antenatal corticosteroids leads to substantial reduction in the incidence of periintraventricular hemorrhage^17,28-30^ and its severe forms^35^ that produce important neurological sequelae among the survivors.^36^ In addition to this, the cardiovascular stabilization promoted by corticosteroids and the modifications in renal function are essential for the extra-uterine survival of extremely premature infants.^37^ Antenatal corticosteroid therapy exercises an influence on practically all the organs and systems of the fetus and avoids or eases the complications most commonly associated with prematurity.

In the United States, the use of antenatal corticosteroids on newborns with a birth weight of less than 1,500 g increased from levels close to 20% at the start of the 1990s to 60% in 1995.^38^ At present, antenatal corticosteroid utilization covers around 70% of the newborns of very low weight at 14 American centers that belong to the NICHD Neonatal Research Network.^39^ In Latin America, the few data available indicate that corticosteroids are used on around 30% of the pregnant women with premature deliveries.^40-42^ In Brazil, there are some reports of antenatal corticosteroid use for inducing fetal lung maturation. In a study covering seven public maternity hospitals in Rio de Janeiro, the antenatal corticosteroid utilization was 4% and 2% for patients with gestational ages of under 34 and up to 36 weeks, respectively.^2^ Another survey was done on premature infants with birth weights of under 1,500 g, born in eight neonatal intensive care units at university hospital referral facilities in various Brazilian states, during the period from March 1998 to December 1999. This showed an average utilization rate for antenatal corticosteroids of 29%, varying between 10% and 39% across the eight facilities.^43^ In other words, the scarce data available with respect to the frequency of antenatal corticosteroid utilization in our environment reveal that this therapeutic strategy has been underused, in the light of its potential benefits.

In view of the potential benefits of antenatal corticosteroid therapy and the sparseness of publications regarding its use in Brazil, there was a need for the present study. Its general objective was to evaluate the effectiveness of antenatal corticosteroids on newborns with gestational ages of less than 34 weeks. The specific endpoints were to evaluate whether antenatal corticosteroids diminished the incidence of respiratory distress syndrome and the occurrence of intra-hospital deaths among Brazilian premature infants.

## METHODS

### Design, setting and sample

The present study was formed by a retrospective cohort based on an analysis of the records of patients with a gestational age of less than 34 weeks and their respective mothers. These patients were born during the period from January 1988 to December 1998, in Hospital São Paulo, of Universidade Federal de São Paulo. The research protocol was approved by the Committee for Ethics in Research of the institution.

### Inclusion and exclusion criteria

For the evaluation of antenatal corticosteroid use and the occurrence of respiratory distress syndrome and intra-hospital deaths, patients that fulfilled the following criteria were included in the study:

- Newborns whose mothers were exposed to antenatal corticosteroid therapy, in accordance with the individual decision of the obstetrician;
- Newborns whose mothers were not exposed to any dose of antenatal corticosteroids. The neonates in this group were chosen to match with exposed neonates, according to sex (same as the exposed neonate), gestational age (within about two weeks), birth weight (within approximately 250 g) and the time of the birth (within up to 100 days of the birth of the exposed newborn).

The criteria for excluding the patient from the population studied were:

- Presence of major congenital malformations^44^
- Use of corticosteroids by the pregnant mother for a purpose other than fetal maturation
- Antenatal corticosteroid use occurring more than seven days before the delivery.

### Variables and Data obtained

The records of the mothers and their newborns were researched to obtain the following data.

***Demographic variables for the mother:*** age and race; pre-existing clinical and obstetric diseases in the present pregnancy; presence of prenatal care (more than four visits); presence of premature delivery labor; need for tocolytics; presence of prolonged rupture of membranes (more than 24 hours) and chorioamnionitis.^45^

***Steroid therapy for the mother:*** an analysis was made of the drugs utilized (betamethasone, dexamethasone or hydrocortisone), the number of doses applied, the number of hours or days between the first dose and the time of delivery, and the number of courses received by the pregnant woman. A complete course was defined as the use of two doses of betamethasone or four doses of dexamethasone or hydrocortisone, between 24 hours and 7 days prior to delivery. An incomplete course was defined as one dose of betamethasone or less than four doses of dexamethasone or hydrocortisone within seven days prior to delivery or, when any dose of corticosteroid was utilized during the 24 hours that preceded the delivery. Multiple courses were considered to have occurred when two or more complete courses of antenatal corticosteroids were administered to the patient.

***Demographic variables for the newborns:*** data were obtained on the need for resuscitation in the delivery room,^46^ the Apgar score^47^ at the first and fifth minute of life, sex, birth weight and gestational age, determined from obstetric data or from a neonatal examination,^48-50^ as well as the classification of the newborn^51^ and presence of twinning.

***Clinical neonatal outcomes:***

- Respiratory distress syndrome: respiratory distress starting within six hours of life and typical radiological findings.^52,53^
- Intra-hospital deaths: an analysis was made of the age when death occurred and its immediate cause according to the neonatologist in charge of the patient or the pathologist who did the necropsy.
- Use of surfactants: among the patients with a diagnosis of respiratory distress syndrome, the number of doses and type of exogenous surfactant utilized were verified.
- Mechanical ventilation: the need for and duration of mechanical ventilation were evaluated.
- Bronchopulmonary dysplasia: defined as oxygen need at 28 days of life, with fixed radiological alterations.^54^
- Peri-intraventricular hemorrhage: the method utilized for its diagnosis was head ultrasonography, performed at the bedside, generally on the fourth, tenth and thirtieth days of life, and classified according to Papile et al.^55^
- Necrotizing enterocolitis: the presence of a clinical-radiological or surgical diagnosis of necrotizing enterocolitis was verified, and classified according to Walsh & Kliegman.^56^

### Statistical Analysis

The chi-squared ^57^ or Fisher Exact^57^ tests were applied for comparing category data between the antenatal corticosteroid and nonantenatal corticosteroid groups. Student's t test^58^ was utilized for the numerical data. Because the length of stay in the hospital, the duration of mechanical ventilation and the number of doses of surfactant did not present normal distributions, these were compared between the groups by the Mann-Whitney test.^59^ The comparison between clinical outcomes for the two groups studied was made by the calculation of the odds ratio (OR) and its confidence interval (CI).^57^ In all the tests, the level of significance was set at 0.05.

For the paired analysis of the patients with and without antenatal corticosteroids during the period studied, an initial analysis was done on 244 newborns whose mothers had received corticosteroids for fetal maturation not more than seven days before the delivery. However, it was only possible to match up 205 newborns (84%). The remaining 39 neonates, for whom it was not possible to find a matching patient not exposed to antenatal corticosteroids within the criteria established above, were excluded from this first analysis. The power of the sample (205 newborns exposed to antenatal corticosteroids and 205 not exposed) was calculated for the occurrence of respiratory distress syndrome and intra-hospital deaths as 99% and 76%, respectively.

To analyze what the role of antenatal corticosteroids was, among the various peri-natal factors that could have interfered in the occurrence of respiratory distress syndrome and intra-hospital death, logistic regression models were made,^60^ utilizing the Statistical Package for the Social Sciences (SPSS) software, version 8.0. This study included the 244 patients whose mothers utilized antenatal corticosteroids, along with the 205 who did not utilize this therapy, for the period from January 1988 to December 1998.

## RESULTS

During the period of the study, 1,051 premature infants were born with a gestational age of less than 34 weeks, after excluding those with major congenital anomalies. Of these 1,051 patients, it was not possible to recover the records of 28 of the mothers, and so the review was done on 1,023 (99.5%) of the records, for verifying the inclusion and exclusion criteria of the study. Of the 1,023 records, it was observed that 258 mothers made use of corticosteroids to induce fetal maturation not more than seven days before the delivery and of these, 244 records (95%) of the respective newborns were recovered.

The neonates forming the group that did not undergo antenatal corticosteroid therapy were chosen from among the 1,023 patients whose records were recovered, excluding the 258 newborns who utilized antenatal corticosteroids. For this, as described earlier, the neonates exposed to antenatal corticosteroids were paired with non-exposed neonates. The majority of the 39 newborns for whom it was not possible to find a pair were born in the years 1997 and 1998, when the rate of utilization of antenatal corticosteroids by the obstetrics team increased. We emphasize that the use of antenatal corticosteroids went on increasing gradually, varying from 5-10% in the years 1988 and 1989 and reaching half of the pregnant mothers in premature delivery labor in the years 1997 and 1998.

The great majority of the mothers (241 out of 244, i.e. 99%) received betamethasone for the induction of fetal maturation. With regard to the number of doses of betamethasone, it was observed that 50 patients (20%) received only one dose, 125 (51%) two doses and 69 (28%) more than two doses. With regard to the interval between the administration of the first dose of the medication and the delivery, 53 pregnant women (22%) received the steroid less than 24 hours before the delivery and 191 (78%) between 24 hours and seven days prior to delivery. At least one complete course of antenatal corticosteroids was accomplished by 184 pregnant women (75%), and 60 (25%) received one incomplete course of treatment. Among those who received the complete course, a single course of antenatal corticosteroids was used on 135 (73%) and multiple courses on 49 (27%).

The comparison of the maternal data between the newborns who made use of the antenatal therapy and those who did not is to be found in Table 1. In the group exposed to antenatal corticosteroids, there was a higher frequency of pregnant women presenting pregnancy-induced hypertension, a greater number of cesarean sections, a greater need for tocolytics and a longer length of stay in the hospital.

**Table 1:** Demographic characteristics of the mothers and newborns exposed or not exposed to antenatal corticosteroids, expressed by number of patients (percentage) or mean (standard deviation)

	EXPOSED n = 205		NOT EXPOSED n = 205		p
**Maternal characteristics**					
Age (years)	27	(7)	26	(6)	0.141**
White race	133	(65%)	113	(55%)	0.055*
Prenatal care	132	(64%)	110	(54%)	0.034*
Pregnancy-induced hypertension	64	(31%)	37	(18%)	0.002*
Diabetes	11	(5%)	5	(2%)	0.202*
Use of tocolytics	45	(22%)	14	(7%)	< 0.001*
Rupture of membranes > 24 hours	161	(79%)	151	(74%)	0.297*
Chorioamnionitis	11	(5%)	16	(8%)	0.425*
Cesarean section	124	(60%)	77	(38%)	< 0.001*
Length of stay (days)	7	(8)	2	(3)	< 0.001***
Neonatal characteristics					
Weight (grams)	1289	(394)	1290	(389)	0.988**
Gestational age (weeks)	31	(2)	31	(3)	0.184**
Resuscitation †	182	(89%)	191	(93%)	0.167*
Apgar 1	6	(2)	5	(3)	0.009**
Apgar 5	8	(2)	8	(2)	0.097**
Small for gestational age	53	(26%)	34	(17%)	0.029*
Twinning	23	(11%)	26	(13%)	0.760*

* *Chi-squared test*

** *Student's t test*

*** *Mann-Whitney test*

† *Resuscitation: need for free-flow oxygen and/or positive pressure ventilation and/or chest compressions and/or cardiac medications in the delivery room.*

With regard to the demographic characteristics of the newborns who received antenatal corticosteroids and those that did not, Table 1 demonstrates an average weight of 1289.5 g and a gestational age of 31 weeks for the two groups. The Apgar score at one minute was greater for the neonates with antenatal corticosteroids and there was a predominance of newborns that were small for gestational age in the group exposed to corticosteroids.

The comparison of the main clinical outcomes for the neonates who received antenatal corticosteroids or not is demonstrated in Table 2. A significant reduction was noted in the incidence of respiratory distress syndrome in the treated group in relation to the group not exposed to corticosteroids. Intra-hospital death was also significantly reduced among the patients that received antenatal corticosteroids, in comparison to those without the medication. With regard to the secondary outcomes observed in the present study, there was a reduction in the need for surfactant therapy and in the more severe peri-intraventricular hemorrhage. The average number of doses of exogenous surfactant (exposed group: 0.3 doses; non-exposed group: 0.6 doses; p = 0.010) and the average number of days on mechanical ventilation (exposed group: 4 days; non-exposed group: 6 days; p < 0.001) were less among neonates who received antenatal corticosteroid therapy.

**Table 2:** Clinical outcomes for newborns exposed or not exposed to antenatal corticosteroids

	EXPOSED number (%)		NOT EXPOSED number (%)		OR	95% CI
Respiratory distress syndrome	52	(25%)	101	(49%)	0.33	(0.21-0.51)
Need for mechanical ventilation	109	(53%)	128	(62%)	0.68	(0.45-1.03)
Need for surfactant	29	(14%)	63	(31%)	0.37	(0.22-0.62)
Bronchopulmonary dysplasia	26	(13%)	33	(16%)	0.76	(0.42-1.37)
Peri-intraventricular hemorrhage	58	(28%)	70	(34%)	0.67	(0.42-1.07)
Peri-intraventricular hemorrhage III and IV	7	(12%)*	23	(33%)*	0.28	(0.10-0.77)
Necrotizing enterocolitis	19	(8%)	19	(9%)	1.00	(0.49-2.06)
Intra-hospital deaths	40	(20%)	66	(32%)	0.51	(0.38-0.82)

*The comparisons between groups are expressed by the odds ratio and 95% confidence interval (CI).*

* *The percentage refers to the number of patients who presented the severe condition in relation to the number of patients with peri-intraventricular hemorrhage.*

With regard to the factors that influenced the occurrence of respiratory distress syndrome in the population studied, the following were significant:

- Use of antenatal corticosteroids: this was a significant protective factor against respiratory distress syndrome (OR: 0.278; 95% CI: 0.177-0.437). In the cohort studied, the adjusted chance that a premature infant not receiving antenatal corticosteroids would develop respiratory distress syndrome was 3.7 times greater than for a neonate exposed to the medication
- Gestational age: the chance that a child in this cohort would develop respiratory distress syndrome diminished by 1/0.75 (1.33) for each increase of one week in gestational age at the time of its birth (OR: 0.758; 95% CI: 0.660-0.869)
- Weight: the chance that a newborn in the present study would develop respiratory distress syndrome diminished by 1/0.99 (1.01) for each increase of one gram in its birth weight (OR: 0.999; 95% CI: 0.998-0.999)
- Apgar score of less than seven at the fifth minute: the risk that a newborn with a gestational age of less than 34 weeks and an Apgar at the fifth minute of less than 7 would present respiratory distress syndrome was 2.48 times greater than for neonates with an Apgar at the fifth minute of greater than 7 (OR: 2.485; 95% CI: 1.370-4.506).

With regard to the factors that influenced the number of intra-hospital deaths in the population studied, the following were significant.

- Prenatal care: the chance that a neonate in the population studied whose mother did not have prenatal care would die in hospital was 1/0.503 (1.98) times the chance for a child whose mother had four or more prenatal care visits (OR: 0.503; 95% CI: 0.294-0.860).
- Gestational age: the chance that a child in the study would die diminished by 1/ 0.844 (1.18) for each additional week of gestational age at the time of birth (OR: 0.844; 95% CI: 0.736-0.968).
- Birth weight: the chance that a child in this cohort would die diminished by 1/ 0.9989 (1.0011) for each addition of one gram to the birth weight (OR: 0.998; 95% CI: 0.998-0.999).
- Respiratory distress syndrome: the presence of respiratory distress syndrome in the population analyzed increased the chance of intra-hospital death by a factor of 1.68 (95% CI: 1.048-2.707).
- Necrotizing enterocolitis: this also constituted a significant risk factor, increasing the risk of death almost threefold (OR: 2.594; 95% CI: 1.263-5.402).
- Mechanical ventilation: the chance that a newborn in this cohort who needed mechanical ventilation would die in hospital was 4.3 times greater than for infants who did not need ventilatory support (OR: 4.318; 95% CI: 2.002-9.309).
- Apgar at fifth minute of less than 7: its presence caused an almost fivefold increase in the chance that a newborn in this cohort would die (OR: 4.709; 95% CI: 2.558-6.432).

## DISCUSSION

A retrospective cohort study was proposed because the research would take in a period of ten years during which technological advances and improvements in the conditions of assistance to neonates took place. Because of these advances, the comparison of newborns who received antenatal corticosteroid therapy or not some years ago with those born in more recent years would not be possible. The study would need to be retrospective, because, as the benefits of antenatal corticosteroid therapy on the evolution of neonates had already been demonstrated in the literature, it would not be possible to submit patients to a prospective study.

With regard to the rate of utilization of antenatal corticosteroids at Hospital São Paulo/Universidade Federal de São Paulo during the period studied, the relatively frequent use of this medication can be explained by the fact that this hospital provides a referral service for high-risk pregnant women and cases of elective prematurity. That is, 40% to 50% of all the premature births there correspond to planned prematurity due to maternal or fetal problems.

Betamethasone was the medication of choice for 99% of the patients. Because betamethasone and dexamethasone present similar structures and longer half-lives and cross the placenta in a biologically active form, they are the drugs most used in antenatal therapy.^18^ At present, betamethasone is the most recommended drug for the induction of fetal maturation^61^ because dexamethasone has been associated with an increase in the risk for periventricular leukomalacia.^62^ The corticosteroid doses prescribed by the obstetricians also followed the general guidelines.^4,17,18^ In this way, the findings from the present study in relation to the utilization strategy for antenatal corticosteroids were similar to those in the international literature. This being so, the clinical results to be expected also ought to be similar to those described for developed countries.

With regard to the maternal characteristics (Table 1), it was noted that, in the group exposed to the antenatal therapy, there was a greater number of pregnant women with prenatal care, a greater frequency of pregnancy-induced hypertension and cesarean sections. These results reflect the fact that antenatal corticosteroids are more frequently administered in our context to women with adequate prenatal care who present problems during the pregnancy that often result in anticipated delivery. In addition to this, the number of patients who utilized tocolytics was greater in the group exposed to antenatal corticosteroids, with this finding being expected and in agreement with data in the literature.^33^ This is because the women to whom tocolytics are administered for the inhibition of premature delivery labor end up receiving the associated corticosteroid therapy.

In relation to the newborns (Table 1), more infants that were small for the gestational age were found in the group that received antenatal corticosteroids, which may have contributed towards a lower occurrence of respiratory distress syndrome among the treated patients. As the patients who received antenatal corticosteroids were those with complicated pregnancies, the presence of a higher frequency of neonates that were small for the gestational age would be expected. In older studies, it was demonstrated that fetal lung maturation was accelerated in the presence of maternal clinical situations that led to chronic stress, thus promoting a lower incidence of respiratory distress syndrome.^63^ On the other hand, later data indicated that when neonates of the same gestational age are compared, the small ones do not show advantages in terms of a reduction in the incidence of respiratory distress syndrome. Moreover, the small ones present a greater need for mechanical ventilation and more prolonged hospital stay, as well as increased intra-hospital mortality.^64,65^

In the comparison of the Apgar score at the first and fifth minutes between the patients who received antenatal corticosteroids and those who did not, the newborns exposed to corticosteroid therapy presented a significantly greater average Apgar score at the first minute of life than those who were not. The higher Apgar scores for patients who received antenatal corticosteroids probably reflect the role that this class of medication plays in the cardiovascular and respiratory stabilization of premature neonates. Studies on animals have shown evidence of the effect of corticosteroids on the mechanisms for adaptation to extra-uterine life. Stein et al.^66^ demonstrated, on premature lambs, that corticosteroids led to an improvement in ventilatory, circulatory and metabolic functions of the animals treated, which was related to the increase in the adenyl-cyclase activity of the myocardium. Corticosteroids are also thought to promote an increase in the expression of adrenergic receptors in vessel walls and the myocardium, which assist in cardiocirculatory stabilization at birth.^24^ This being so, the higher average Apgar score at the first minute for patients exposed to antenatal corticosteroid therapy, observed in this study, may represent a greater capacity among the premature infants for adaptation to the extra-uterine environment.

In relation to the main clinical outcomes for the newborns (Table 2), it was observed that in this study antenatal corticosteroids had a significant protective role, reducing the occurrence of respiratory distress syndrome in the population studied by more than 50%. In the latest revision of Crowley's meta-analysis,^30^ the use of antenatal corticosteroids led to a significant reduction in the incidence of respiratory distress syndrome, with the value of the odds ratio (OR 0.53; 95% CI: 0.44-0.63) being similar to what was found in the present study. The large multicenter research projects that have been done to evaluate the effect of exogenous surfactants on the evolution of premature infants, which retrospectively analyzed the data on the use of antenatal corticosteroids in important American and Canadian neonatal networks, also demonstrate the benefits of corticosteroid therapy.^34,67-69^ In a gathering-together of all these studies,^70^ involving data on more than 35,000 newborns, it was demonstrated that antenatal corticosteroids significantly reduced the incidence of respiratory distress syndrome, with a non-adjusted odds ratio of between 0.43 and 0.87.

Nonetheless, even with this positive result, the diminution of the incidence of respiratory distress syndrome in neonates exposed to antenatal corticosteroid therapy in our environment has to be analyzed in conjunction with other factors that could be interfering in the results. In the final logistic regression model for the dependent variable "respiratory distress syndrome", the use of antenatal corticosteroids persisted as a significant protection factor against the occurrence of respiratory distress in the population analyzed. This finding reinforces the protective role of antenatal corticosteroids in relation to respiratory distress syndrome in our population. Taking into account the high annual birth rates for low-birth-weight and premature infants in Brazil, and considering that respiratory problems and respiratory distress syndrome represent around 50% of the deaths during the neonatal period,^1^ a more widespread use of corticosteroid therapy could have a large impact on specific neonatal mortality. In the logistic regression model for the variable of respiratory distress syndrome response, it was also observed that greater gestational age and higher birth weight had a significant protective role against the occurrence of respiratory distress syndrome.

These data confirm what is shown in the literature: low birth weight and younger gestational age increase the risk of respiratory distress syndrome and it is in this population that antenatal corticosteroids exercise their protective effect.^61^ This occurs even under technological conditions for the care of extremely premature infants that are far from those found in developed countries.

It was also observed that asphyxia (Apgar at the fifth minute of less than 7) was a significant risk factor for the occurrence of respiratory distress syndrome in the population studied. In these circumstances, antenatal corticosteroids would also exercise a protective role in cardiorespiratory stabilization at birth^66^ and their contribution towards successful adaptation to extra-uterine life would indirectly influence the frequency of respiratory distress syndrome, diminishing its occurrence.

The incidence of intra-hospital death among patients who utilized antenatal corticosteroids and those who did not was also significantly lower in the treated group. Crowley's meta-analysis,^30^ which included those born before and after exogenous surfactants became available, found an odds ratio of 0.60 (95% CI: 0.48-0.75) for neonatal mortality in relation to the utilization of antenatal corticosteroids. However, in this meta-analysis, the impact of antenatal corticosteroid therapy was lower among patients born soon after 1980 (OR: 0.78; 95% CI: 0.54-1.12), when the mortality due to respiratory distress syndrome had not yet become so significant and other factors may have exercised greater influence on deaths among premature infants in the developed countries.

In the present study, the fact that the magnitude of the protective role of antenatal steroids is close to that found during the period before exogenous surfactants were available in developed countries reflects the neonatal pattern ruling in Brazil: respiratory affections are still an important cause of death among newborns.^1^ However, when the use of the medication was analyzed together with other factors that could interfere in neonatal mortality, antenatal corticosteroids did not persist as a protective factor in the cohort studied. It is known that antenatal corticosteroids increase the survival of neonates that previously would not have had the chance of survival. As a consequence, such patients come to present complications resulting from the prolonged hospital stay, such as neonatal sepsis, necrotizing enterocolitis and chronic lung disease, among others. Such complications, in their turn, may contribute to neonatal mortality. This being so, when all these factors are analyzed together, the protective role of corticosteroids in the survival of premature infants may not be evident.

In the population studied, even though this study was conducted in a tertiary-level hospital, the mortality of premature infants stratified by birth weight was significantly higher than that reported in developed countries. Consequently, the influence of factors like the absence of prenatal care, the presence of perinatal asphyxia and prenatal and post-natal infections, among others, may be more important than the impact of the antenatal corticosteroid therapy on neonatal mortality.

This speculation is, in a way, confirmed in the present study, in observing the risk factors for the occurrence of intra-hospital death in the cohort analyzed. These were the presence of respiratory distress syndrome and necrotizing enterocolitis, the need for mechanical ventilation and the perinatal asphyxia. It is worth emphasizing that the presence of respiratory distress syndrome increased the chance that a neonate from the population studied would die, by a factor of 1.68. This fact demonstrates that, even in the Neonatal Unit of Hospital São Paulo, a tertiary-level university facility that routinely uses mechanical ventilation and has had exogenous surfactants available since 1992, this respiratory affection still represents an important risk factor for intra-hospital mortality among premature neonates.

Added to this observation is the finding that the need for mechanical ventilation increased the chance that a neonate in the population studied would die, by a factor of 4.3. Antenatal corticosteroids, through the pulmonary maturation that they provide, prevent the appearance of respiratory distress syndrome and thereby provide a diminution of the need for and length of mechanical ventilation, as well as reducing the doses of exogenous surfactants utilized. In this way, corticosteroids are thought to indirectly exercise a protective effect on intra-hospital neonatal mortality resulting from respiratory distress syndrome and the use of mechanical ventilation.

In the present study, the incidence of peri-intraventricular hemorrhage as a whole was not reduced in the group treated with antenatal corticosteroids, contrary to data in the literature, which demonstrates a significant diminution in the overall incidence of peri-intraventricular hemorrhage.^17^ Nevertheless, as this project covered a period of ten years, during which head ultrasonography was not routinely available initially, the sample studied would not have had sufficient statistical power to demonstrate a significant difference between the groups. Even so, a significant reduction was found in the severe forms of peri-intraventricular hemorrhage, or in other words, hemorrhages of grades III and IV, among the patients treated with antenatal corticosteroids. It has to be remembered that the more advanced grades of peri-intraventricular hemorrhage are the most worrying, as these are associated with high rates of mortality and neurological sequelae among the survivors.^36^

The data in the literature also demonstrate a diminution in the incidence of severe forms of peri-intraventricular hemorrhage when antenatal corticosteroids are used. Shankaran et al.,^35^ in a survey of 4,665 neonates of very low birth weight, showed that complete courses of antenatal corticosteroids were protective against the occurrence of severe forms of periintraventricular hemorrhage (OR: 0.39; 95% CI: 0.27-0.57), similar to that found in our study. The data presented here reinforce the protective power of antenatal corticosteroids regarding the occurrence of severe and worrying forms of peri-intraventricular hemorrhage.

The reduction in the incidence of respiratory distress syndrome, the number of deaths, the time on mechanical ventilation, the number of doses of exogenous surfactant utilized and the incidence of severe forms of peri-intraventricular hemorrhage have, as a consequence, a lower hospital cost for the premature infant admitted in an intensive care unit. In this way, in countries with less availability of financial resources for the treatment of this type of patient, like in Brazil, antenatal corticosteroids represent a low-cost and effective resource for the reduction of neonatal morbidity and mortality.
